# Is the medical financial assistance program an effective supplement to social health insurance for low-income households in China? A cross-sectional study

**DOI:** 10.1186/s12939-017-0638-3

**Published:** 2017-08-01

**Authors:** Kai Liu, Jing Yang, Chunling Lu

**Affiliations:** 1 Department of Social Security, School of Labor and Human Resources, Renmin University of China, 59 Zhongguancun Street, Haidian District, Beijing, 100872 China; 2 School of Sociology and Population Studies, Renmin University of China, Beijing, China; 3 Division of Global Health Equity, Brigham & Women’s Hospital, Boston, MA USA; 4 Department of Global Health and Social Medicine, Harvard Medical School, Boston, MA USA

**Keywords:** China, Medical financial assistance, Cash aid, Social health insurance, Catastrophic health expenditure

## Abstract

**Background:**

China uses both social health insurance (SHI) programs and a medical financial assistance (MFA) program to protect the poor from illness-induced financial risks. The MFA provides a dual benefit package targeting low-income families: subsidizing these families’ participation in SHI programs, and providing cash aid to protect them from catastrophic health expenditure (CHE). This study aims to investigate: (1) the association between MFA subvention for SHI enrollment and SHI enrollment; (2) the association between MFA cash aid and CHE; and (3) the association between SHI enrollment and CHE in low-income households in China.

**Methods:**

Using nationally representative data from a comprehensive survey of low-income households in 2014, we construct an estimate of CHE based on out-of-pocket health spending data. Controlling for other covariates, we estimate the three associations using a three-level logistic model.

**Results:**

The MFA program subsidizes 50.1% of low-income households to aid their enrollment in SHI programs and provides cash aid to 24.1% of these households. Multilevel logistic analysis reveals that MFA subvention has no significant association with low-income households’ SHI enrollment, that MFA cash aid has no significant association with CHE, and that full SHI enrollment is inversely associated with CHE status.

**Conclusions:**

The MFA program is currently not an effective supplement to SHI programs in China in terms of promoting SHI enrollment and providing financial risk protection. The Chinese government needs to invest more funds to expand further low-income household enrollment in SHI programs and to widen the benefit package of MFA cash aid.

**Electronic supplementary material:**

The online version of this article (doi:10.1186/s12939-017-0638-3) contains supplementary material, which is available to authorized users.

## Background

Poverty has been redefined as encompassing not only material deprivation but also low achievements in health, education, and so on [1]. There is a vicious cycle linking poverty and poor health, in that the poor are particularly vulnerable to financial risks caused by serious illness and high out-of-pocket expenditure (OPE) on health, which may further worsen their health [2, 3]. A common measure of the financial risks related to illness is catastrophic health expenditure (CHE) [4, 5]. CHE is defined as a household’s OPE exceeding a substantial fraction of the household’s total expenditure or capacity to pay [4–9]. Previous studies have found that the percentage of households with CHE in China was 9.9% in rural areas in 2001 [10], 12.9% in 2011 [9], and 13.0% in 2012 [11]. In addition, the percentage of households with CHE is region- and socio-demographic characteristic-specific. It is significantly higher in the central and western regions of China, such as Chongqing and Shaanxi Province, than in eastern regions like Shanghai and Shandong Province [10–14]. Undeveloped areas, rural families, households with older adults and chronically ill members, and low-income households are more likely to incur CHE and impoverishment due to health expenditure [12, 15, 16].

Investing in health services is a rational choice for health equity promotion and poverty reduction strategies in developing countries [17]. The World Health Organization (WHO) advocates “pro-poor” health policies and has launched global advocacy, regional initiatives, and direct support for developing countries implementing such health policies [18]. Countries have implemented various health policies to prevent poor families from falling into medical impoverishment, out of which social health insurance (SHI) and medical financial assistance (MFA) programs are most often adopted. SHI, as a financing approach for mobilizing funds and pooling risks, provides health insurance to contributing members and their dependents and often requires mandatory contributions [19, 20]. MFA provides financial aid to cover the poor’s medical spending directly or to assist them to participate in health insurance programs [21, 22]. Many low- and middle-income countries, such as Turkey, Indonesia, Colombia, and Mexico, have used SHI programs to provide mandatory health service coverage for vulnerable people [20, 23–26]. Countries, such as Georgia and many sub-Saharan African countries, have established MFA programs for the poor who have experienced serious illnesses [21, 22]. Other countries, such as India, Rwanda, Lao People’s Democratic Republic, Vietnam, and Senegal, have developed community-based health insurance to protect the enrollees from medical impoverishment [27–33]. The effects of SHI and MFA programs vary in these countries. SHI enrollment accompanies high OPE in Turkey [23], and reduces OPE in Indonesia, Colombia, and Mexico [24–26]. MFA significantly increases medical care utilization by the poor in Georgia [21], while it has low coverage of enrollment among the poor in sub-Saharan African countries [22].

China uses both SHI program and a MFA program to protect the poor from the financial risks of illness. The SHI system in China, introduced in the first decade of the twenty-first century, is targeted at all types of residents. It comprises three government-run schemes (see Table 1) and reached universal coverage at the beginning of the 2010s [34]. The MFA program in China, targeting low-income households, is used as a supplement to SHI programs and provides extra financial assistance to low-income households in addition to SHI. A low-income household in this study is defined as a household where income or assets are officially identified as lower than a certain county-specific criterion. It includes four types of households: households enrolled in the Minimum Living Standard Scheme (MLSS), extremely poor residents, households with a monthly income of between 100% and 120–150% of the local MLSS line, and other vulnerable persons identified by local county government [35–40].

**Table 1:** Policy design of the MFA program and SHI programs in China

	Medical Financial Assistance (MFA)	Social Health Insurance (SHI)
Policy implementation	2003: The Ministry of Civil Affairs piloted the MFA in a few rural areas; 2005: The Ministry of Civil Affairs piloted the MFA in a few urban areas; 2009: The MFA reached universal coverage across the whole country; 2013: The Ministry of Finance and the Ministry of Civil Affairs required localities to coordinate the management of SHI programs and the MFA, aiming to provide one-time reimbursement to target households; 2014: The State Council enacted the Draft Decree on Social Assistance, legitimizing the MFA as an indispensable part of social assistance programs; 2015: The State Council ruled that the MFA gives priority to households with serious illnesses.	1998: The State Council established the Urban Employee Basic Medical Insurance (UEBMI); 2003: The Ministry of Health, the Ministry of Finance, and the Ministry of Agriculture established the New Cooperative Medical Scheme (NCMS); 2007: The State Council established the Urban Resident Basic Medical Insurance (URBMI); 2009: The Communist Party of China Central Committee and the State Council launched a new health care reform; 2010: The three SHI programs reached universal coverage across the whole country. 2016: The State Council decided to integrate the URBMI with the NCMS.
Targets	(1) Households enrolling in the Minimum Living Standard Scheme (MLSS). The MLSS is a national social assistance program and targets the poorest households where monthly or yearly income is lower than a certain criterion. Unlike the national unified poverty line [2300 Chinese Yuan (USD372) in 2011 and 3000 Yuan (USD488) in 2016] that was regulated by the State Council Leading Group Office of Poverty Alleviation and Development to facilitate county-based poverty alleviation, the MLSS, run by the Ministry of Civil Affairs, is a household-based cash aid program. Its criterion of eligibility is county-specific in most areas and province-specific in some others. In 2016, the criterion in Shanghai was a monthly income per person of 880 Yuan (USD143) for a household, the highest in any province. An MLSS applicant must be reviewed through a complicated means-test of his/her household’s monetary income, basic living needs, and other household characteristics such as labor capacity and severe illnesses. The difference between the estimated income of an MLSS household and the local MLSS criterion is paid to the household. (2) Extremely poor residents (EPR, including: “Sanwu,” urban residents with no income, labor capacity, or caregivers; “Wubao,” rural residents with no income, labor capacity, or caregivers; “Tekun,” households defined as extremely poor by the Draft Decree on Social Assistance). (3) Low-income families not enrolled in the MLSS (LIF, identified by local government; the criterion is usually a monthly family income of between 100% and 120–150% of the local MLSS line). (4) Persons who are identified by county government.	(1) The UEBMI provided mandatory coverage to urban employees. (2) The URBMI provided voluntary coverage to urban residents without formal employment. (3) The NCMS provided voluntary coverage to rural residents.
Benefit package	Dual benefit package: (1) Subvention for SHI enrollment. Target households are subsidized for their enrollment in SHI programs. (2) Cash aid. Members of target households can apply for MFA cash aid from the county Bureau of Civil Affairs if their OPE exceeds the thresholds of the MFA. If they are enrolled in a SHI scheme, MFA cash aid is provided as a proportion of their OPE; if not, the MFA cash subsidy is provided as a proportion of their total medical expenditure.	Covers both outpatient and inpatient services and provides reimbursement to patients immediately or afterwards.
Funding sources	Raised from (1) government budget, (2) lottery welfare fund, and (3) society donations; County government normally sets up a special and independent MFA account within the SHI system, manages all funds uniformly, and takes full responsibility for its activities.	Raised by a special SHI agency or taxation agencies by collecting premiums; Premium for the UEBMI is contributed by individual employees and employers; Premium for the URBMI and the NCMS is contributed by individual residents and the government.

Sources: See references [34], [35–41]

The MFA provides a dual benefit package for low-income families: subsidizing their enrollment in a SHI program (MFA subvention for SHI enrollment), and providing cash aid to eligible households to reimburse their medical expenditure (MFA cash aid) [41, 42]. The MFA pays part (partial MFA subvention, over 50% of premiums in most provinces) or all (full MFA subvention, 100% of premiums) of the premium (120 Chinese yuan [USD19.5] per household member in 2016) needed to enroll in a SHI for members of eligible low-income households. Those households obtaining partial MFA subvention must pay the remaining premium themselves. In rural areas, the MFA subsidizes eligible households’ enrollment in the New Cooperative Medical Scheme (NCMS), while in urban areas it subsidizes participation in the Urban Resident Basic Medical Insurance (URBMI). In practice, members in some households choose not to enroll in the two insurance programs after considering their affordability, health status, or employment conditions. As a result, low-income households may have all (full SHI enrollment), part (partial SHI enrollment), or none (no SHI enrollment) of their members enrolled in the insurance programs. Low-income households with a SHI enrollment may have most (more than 50% in practice) of their medical payments covered by SHI programs; if their OPE exceeds the thresholds regulated by the MFA, they can apply for cash aid from local authorities. A detailed chronology of policy design, targets, benefit packages, and funding sources for the MFA and SHI are presented in Table 1. During practical implementation, the policy design of the MFA (including thresholds, reimbursement rates, ceilings, and limitations on the eligibility for various illnesses and medical care services) varies across provinces and counties. For example, the thresholds for serious illnesses range from over USD100 to about USD5,000, while the cash aid for these illnesses accounts for 30–100% of OPE across provinces (see Additional file 1: Table S1).

The Chinese government launched the MFA to achieve two aims: (1) promoting SHI enrollment among the poor; and (2) improving the poor’s access to care and financial risk protection [36–38, 41]. Numerous studies have evaluated the performance of SHI programs in reducing CHE and medical impoverishment [8, 10, 16, 43–47]. Based on a small number of samples collected from a few counties, several studies have investigated the MFA program’s effect on poor families’ utilization of medical care services and their medical expenditure [39, 42, 48]. However, little empirical evidence has been generated on the role the MFA program plays in promoting SHI enrollment and reducing CHE. Given the rapid development and expansion of China’s MFA program, it is necessary and urgent to assess its role in promoting SHI enrollment and financial risk protection to provide evidence for evaluation and health policy development.

**Fig. 1:**
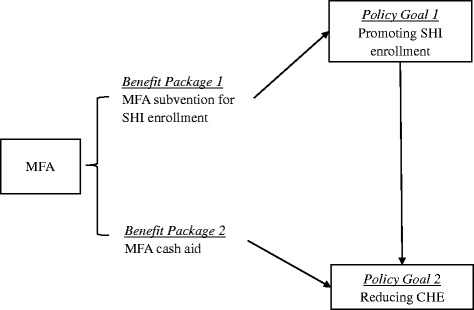
Conceptual framework

Using nationally representative data from a comprehensive survey of low-income households in 2014, this study investigated the role MFA subvention plays in promoting SHI enrollment and the association between MFA cash aid and CHE in China. We also examined the association between SHI enrollment and CHE. Figure 1 illustrates a conceptual framework for this study. To our knowledge, this study is the first policy study that uses a nationally representative dataset to investigate the MFA’s role in facilitating SHI enrollment and protecting low-income households from catastrophic health spending in China.

## Methods

### Data

The data were drawn from a nationally representative dataset collected for the study *Evaluating Social Policy Supporting System for Vulnerable Families in Urban and Rural China* (ESPSS) [49]. The Ministry of Civil Affairs of China launched the ESPSS in 2008 and undertook a program of annual surveys starting in 2012. The data used in this study were collected in 2014.

The ESPSS used a multistage cluster sampling method based on the sampling frame of the Sixth National Population Census of China in 2010. In the first stage, 1500 villages in rural areas and neighborhoods in urban areas were randomly drawn from 29 provinces (Xinjiang, Tibet, Hong Kong, and Macau were not included). In the second stage, seven low-income households were selected from each village and 12 from each neighborhood using quota sampling. Low-income families in the ESPSS data included households enrolled in the MLSS, extremely poor households, households that had made applications for the MLSS (including households that had dropped out of the MLSS), low-income families not enrolled in the MLSS (identified by local government in some provinces), and low-income immigrant households. An official letter about the survey was issued by the Ministry of Civil Affairs to local county-level Bureaus of Civil Affairs to facilitate the collection of information from vulnerable households with the help of local village or neighborhood committees. In 2014, a total of 14,179 low-income families were surveyed, with a response rate of 83%. This study used the information for 11,570 households (7290 urban households and 4280 rural households), excluding 2609 immigrant households.

To answer our study questions, we extracted three sets of subsamples: (1) the Q1 sample for estimating the association between MFA subvention and SHI enrollment. We only used data from households that were either fully enrolled in SHI or uninsured to identify the link; (2) the Q2 sample for examining the association between MFA cash aid and CHE. The MFA provides cash aid to low-income households where the OPE must exceed a county-specific threshold. We therefore only used data from households eligible for MFA cash aid. The detailed strategies on how the threshold was constructed in each county are in Additional file 1: Supplementary Text; and (3) the Q3 sample for estimating the association between SHI enrollment and CHE, which used data from all households in the ESPSS dataset. The final sample size was 9537 households for the Q1 sample, 1521 households for the Q2 sample, and 11,570 households for the Q3 sample.

### Measurement

#### Dependent variables

To evaluate the association between MFA subvention and household SHI enrollment, we used a dummy variable for household SHI enrollment (0 = no SHI enrollment; 1 = full SHI enrollment). To assess the association between MFA cash aid or SHI enrollment and household CHE, we measured CHE using households’ annual OPE data. The ESPSS asked each household to report annual OPE for all its members. It also included questions about households’ total spending, food spending, and household size in 2014. Using the WHO’s approach [4], this study defined a household as having CHE if its annual OPE exceeded 40% of its annual capacity to pay. We measured capacity to pay by subtracting spending on basic subsistence needs from total household expenditure. We calculated basic subsistence needs as the average annual food expenditure of households where food shares were in the 45th and 55th percentiles. We assigned the value “1″ to CHE if the ratio of a household’s annual total OPE to its capacity to pay was 0.4 or above, and “0″ otherwise.

#### Independent variables

To evaluate the association between MFA subvention and household SHI enrollment, we used two dummy variables: full MFA subvention (0 = no MFA subvention; 1 = full MFA subvention) and partial MFA subvention (0 = no MFA subvention; 1 = partial MFA subvention). To examine the association between MFA cash aid and CHE, we used a dummy variable for MFA cash aid (0 = no MFA cash aid; 1 = MFA cash aid). In addition, to assess the association between household SHI enrollment and CHE, we used two dummy variables: full SHI enrollment (0 = no SHI enrollment; 1 = full SHI enrollment) and partial SHI enrollment (0 = no SHI enrollment; 1 = partial SHI enrollment).

#### Other covariates

First, household total medical costs: according to the policy design of the MFA, cash aid is provided only if the OPE or total medical costs of a low-income household exceed the threshold of MFA cash aid, indicating that total medical costs affect the provision of MFA cash aid. In addition, high total medical costs increase the possibility of high OPE and CHE when there is a lack of protection from the social welfare system [50]. Second, health care needs: health care needs may confound the relationship between the MFA/SHI and CHE. We included the number of older adults (aged 65 and older) and the number of children (under five years old), the adjusted health score for household members, the number of people with chronic illnesses, the number of people with serious illnesses, and the number of people needing long-term care. The ESPSS asked the head of each household to report on each members’ health status using a 5-point Likert scale (1 = very good, 2 = good, 3 = normal, 4 = bad, and 5 = very bad). We constructed a variable representing a household’s health score by adding the points from all members in the household. We then regressed the household health score on household’s number of older adults, number of children, number of people with chronic illnesses, and number of people with serious illnesses [51]. We divided the predicted health score by household size to obtain the adjusted household health score. Third, socioeconomic strata variables: socioeconomic strata here involved three dimensions: income, education, and occupation. The ESPSS asked the head of each household to report total household income for the previous year. We used per capita household income, dividing total household income by household size [52]. We also used the number of unemployed and the number of household members with a high school education and above. Fourth, demographic variables: we included urban status (0 = living in rural areas; 1 = living in urban areas) and the number of male members. Summary statistics of these variables are presented in Table 2.

**Table 2:** Summary statistics of variables used in the three multilevel logistic models

	Q1 sample			Q2 sample			Q3 sample		
	*N*	Mean	SD	*N*	Mean	SD	*N*	Mean	SD
CHE	9389	0.50	0.50	1502	0.69	0.46	11,386	0.49	0.50
Household SHI enrollment									
No SHI enrollment	9521	0.28	0.45	-	-	-	11,554	0.23	0.42
Partial SHI enrollment	-	-	-	-	-	-	11,554	0.18	0.38
Full SHI enrollment	9521	0.72	0.45	-	-	-	11,554	0.59	0.49
MFA cash aid	9525	0.23	0.42	1509	0.45	0.50	-	-	-
MFA subvention for SHI enrollment									
No subvention	8527	0.50	0.50	-	-	-	-	-	-
Partial subvention	8527	0.25	0.43	-	-	-	-	-	-
Full subvention	8527	0.25	0.43	-	-	-	-	-	-
Log (total medical cost)	8536	7.03	1.67	1521	8.19	1.08	10,442	7.08	1.68
Urban	9537	0.61	0.49	1521	0.63	0.48	11,570	0.63	0.48
Number of males	9537	1.47	0.91	1521	1.64	0.95	11,570	1.52	0.92
Number of older adults	9537	0.56	0.75	1521	0.65	0.82	11,570	0.56	0.75
Number of children	9537	0.08	0.31	1521	0.10	0.34	11,570	0.09	0.33
Adjusted household health score	9520	3.78	2.06	1519	3.92	1.93	11,551	3.59	1.96
Number of people with chronic illness	9525	0.76	0.82	1519	0.78	0.87	11,557	0.78	0.83
Number of people with serious illness	9530	0.28	0.52	1521	1.14	0.38	11,562	0.30	0.54
Number of people needing long-term care	9535	0.42	0.64	1521	0.70	0.71	11,568	0.44	0.65
Number of people with high school education and above	9537	0.52	0.83	1521	0.64	0.89	11,570	0.60	0.88
Number of unemployed	9537	0.78	0.82	1521	1.02	0.85	11,570	0.80	0.82
Log (per capita household income)	9537	4.11	8.29	1521	4.20	8.18	11,570	4.28	8.09

The Q1 sample was used to assess the association between MFA subvention for SHI enrollment and SHI enrollment (Eq. 1); the Q2 sample was used to examine the association between MFA cash aid and CHE after addressing self-selection bias (Eq. 2); and the Q3 sample was used to analyze the association between SHI enrollment and CHE (Eq. 3)

We converted the value of all expenditure- and cost-related items to the value of the Chinese yuan in 2014 [53]. Next, we transformed the unit of expenditures and costs from Chinese yuan to US dollars, using the 2014 exchange rate of 6.143 [54]. In addition, we took the logarithm on all expenditure- and cost-related variables to render positively skewed distributions normal and to increase the efficiency of the analysis. To families with no medical expenditure (medical expenditure = 0) we assigned an extremely small value, 10^−10^, to facilitate log transformation. Out of 11,570 observations, data for 0.90% of households’ expenditure, 2.43% of households’ food expenditure, and 0.30% of households’ income were missing. We used regression imputation to address the issue of missing data [55, 56]. We regressed these expenditure variables on all independent variables having high correlations with them in a correlation matrix and then replaced the missing values with predictive values.

### Statistical analysis

Using the full sample, we first reported on the percentage of low-income households enrolled in SHI programs and those receiving the MFA subvention, respectively in rural and urban areas. We then described the percentage of households with CHE by household MFA cash aid and SHI enrollment status, respectively across provinces. We also demonstrated the percentage of households receiving MFA cash aid in rural and urban areas.

A multilevel random intercepts model was used to control for potential clustering effects at provincial and county levels [57]. Three log binominal regressions were fitted for the two binary dependent variables, household SHI enrollment and household CHE status, as follows:

1 $$ Logit\left({SHIenrol}_{ijk}\right)={\beta}_{10}+{\beta}_{11}{FullMFAsub}_{ijk}+{\beta}_{12} PartMFAs{ub}_{ijk}+{\boldsymbol{\beta}}_{13}{\boldsymbol{X}}_{\boldsymbol{ijk}}+{\upsilon}_{10k}+{u}_{10jk} $$

2 $$ Logit\left({CHE}_{ijk}\right)={\beta}_{20}+{\beta}_{21} MFAcas{h}_{ijk}+{\boldsymbol{\beta}}_{22}{\boldsymbol{X}}_{\boldsymbol{ijk}}+{\upsilon}_{20k}+{u}_{20jk} $$

3 $$ Logit\left({CHE}_{ijk}\right)={\beta}_{30}+{\beta}_{31}{FullSHIenrol}_{ijk}+{\beta}_{32}{PartSHIenrol}_{ijk}+{\boldsymbol{\beta}}_{33}{\boldsymbol{X}}_{\boldsymbol{ijk}}+{\upsilon}_{30k}+{u}_{30jk} $$

where Logit(*SHIenrol*_*ijk*_) represents the probability of full enrollment in SHI programs by all household members of the *i*th household in the *j*th county and *k*th province; Logit(*CHE*_*ijk*_) represents the probability of CHE for the *i*th household in the *j*th county and *k*th province; *FullMFAsub*_*ijk*_, *PartMFAsub*_*ijk*_, *MFAcash*_*ijk*_ *, FullSHIenrol*_*ijk*_, and *PartSHIenrol*_*ijk*_ represent, respectively, full MFA subvention for SHI enrollment, partial MFA subvention for SHI enrollment, MFA cash aid, full SHI enrollment, and partial SHI enrollment for the *i*th household in the *j*th county and *k*th province; ***β***_***13***_, ***β***_***22***_, and ***β***_***33***_ are vectors of the coefficients for ***X***_***ijk***_, which is a vector of covariates on household characteristics for the *i*th household in the *j*th county and *k*th province; and *ν*_*10k/*_ *ν*_*20k/*_ *ν*_*30k*_ and *μ*_*10jk/*_ *μ*_*20jk/*_ *μ*_*30jk*_ represent between-province random variation and between-county/within-province random variation, respectively.

Total medical cost was controlled for in Eqs 2 and 3 because it may be correlated with SHI enrollment, eligibility for MFA cash aid, and CHE status. It was not included in Eq. 1. In addition, Eq. 1 was estimated with the Q1 sample, the Q2 sample was used for estimating Eq. 2, and the Q3 sample, for Eq. 3.

To test the sensitivity of the results, we used propensity score matching to analyze whether the two groups – the treatment group (receiving MFA cash aid) and control group (not receiving MFA cash aid) – differed significantly in the likelihood of CHE (see Additional file 1: Table S5) [58]. To capture the different scenarios between urban and rural areas, we conducted additional analyses on all descriptive and inferential statistics for urban and rural samples, respectively (see Additional file 1: Tables S2, S3, and S4). Given the high regional variation in economic development found in China, we also explored the impact of region (eastern, central, western, and northeastern regions) on the associations between MFA subvention and SHI enrollment, SHI enrollment and CHE, and MFA cash aid and CHE by including an interaction between region and main independent variables in various models (see Additional file 1: Tables S6 and S7).

## Results

### The role of MFA subvention for SHI enrollment

**Fig. 2:**
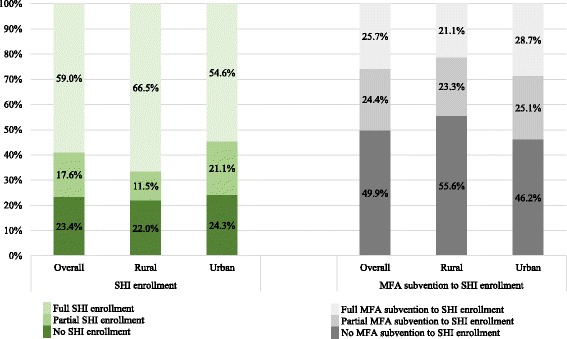
SHI enrollment and MFA subvention for SHI enrollment among low-income households in China

Figure 2 presents SHI enrollment and MFA subvention among low-income households in China. It shows that 23.4% of low-income households (22.0% of rural households and 24.3% of urban households) reported no family members enrolled in SHI programs; 49.9% of low-income households (55.6% of rural households and 46.2% of urban households) received no MFA subvention for SHI enrollment, despite being eligible.

Table 3 shows the association between MFA subvention for SHI enrollment and household SHI enrollment using the Q1 sample. The model fit the data well, with Wald chi2 equal to 258.82 (*p* < 0.001). The results show that MFA subvention for SHI enrollment had no significant association with household SHI enrollment (for partial subvention, OR = 1.015, 95% CI = 0.756–1.364; for full subvention, OR = 1.174, 95% CI = 0.990–1.392). Among other covariates, a household with more members with chronic or serious illnesses and a higher household income was more likely to participate in SHI programs than one with fewer members with chronic or serious conditions and a lower household income. Additional file 1: Table S3 shows that MFA subvention for SHI enrollment had no significant association with household SHI enrollment in either rural or urban areas, except for a significantly positive association between full MFA subvention for SHI enrollment and SHI enrollment in rural areas. Additional file 1: Table S7 indicates no significant association between MFA subvention for SHI enrollment and SHI enrollment across all regions, except for a significantly positive association between full MFA subvention for SHI enrollment and SHI enrollment in the northeastern region.

**Table 3:** Results of multilevel logistic analysis using the Q1 sample: MFA subvention for SHI enrollment and SHI enrollment

Household SHI enrollment (1 = all enrolled; 0 = none enrolled)	Q1 sample (*N* = 8498; Province Group = 29; County Group = 159)	
	OR	95% CI
MFA subvention for SHI enrollment		
No subvention (reference)	reference	
Partial subvention	1.015	(0.756, 1.364)
Full subvention	1.174	(0.990, 1.392)
Urban	0.911	(0.601, 1.382)
Number of males	0.959	(0.819, 1.123)
Number of older adults	1.134	(0.979, 1.315)
Number of children	0.951	(0.712, 1.270)
Adjusted household health score	0.997	(0.957, 1.040)
Number of people with chronic illnesses	1.470***	(1.278, 1.690)
Number of people with serious illnesses	1.265*	(1.049, 1.526)
Number of people needing long-term care	0.999	(0.856, 1.164)
Number of people with high school education and above	1.051	(0.930, 1.188)
Number of unemployed	0.905	(0.793, 1.033)
Log (per capita household income)	1.025***	(1.014, 1.037)

*OR* odds ratio, *CI* confidence interval, *SHI* social health insurance, *MFA* Medical Financial Assistance; * = *p* < 0.05; ** = *p* < 0.01; *** = *p* < 0.001

### The role of MFA cash aid and SHI enrollment

**Fig. 3:**
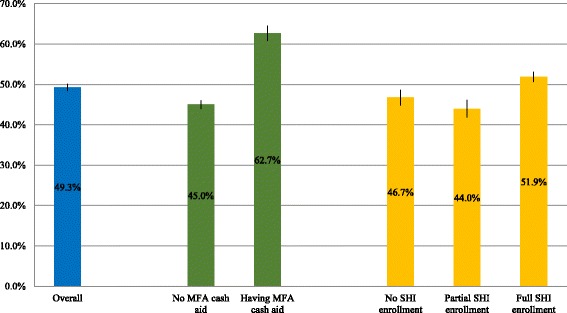
Percentage of low-income households with CHE, by MFA cash aid status and SHI enrollment status

Figure 3 shows the percentage of low-income households with CHE in 2014. Overall, 49.3% of low-income households had CHE status. CHE was more likely to occur among low-income households receiving MFA cash aid (62.7%) and those with full SHI enrollment (51.9%). Additional file 1: Fig. S1 shows the distribution of CHE in urban and rural areas. In general, more rural low-income households had CHE than urban households.

**Fig. 4:**
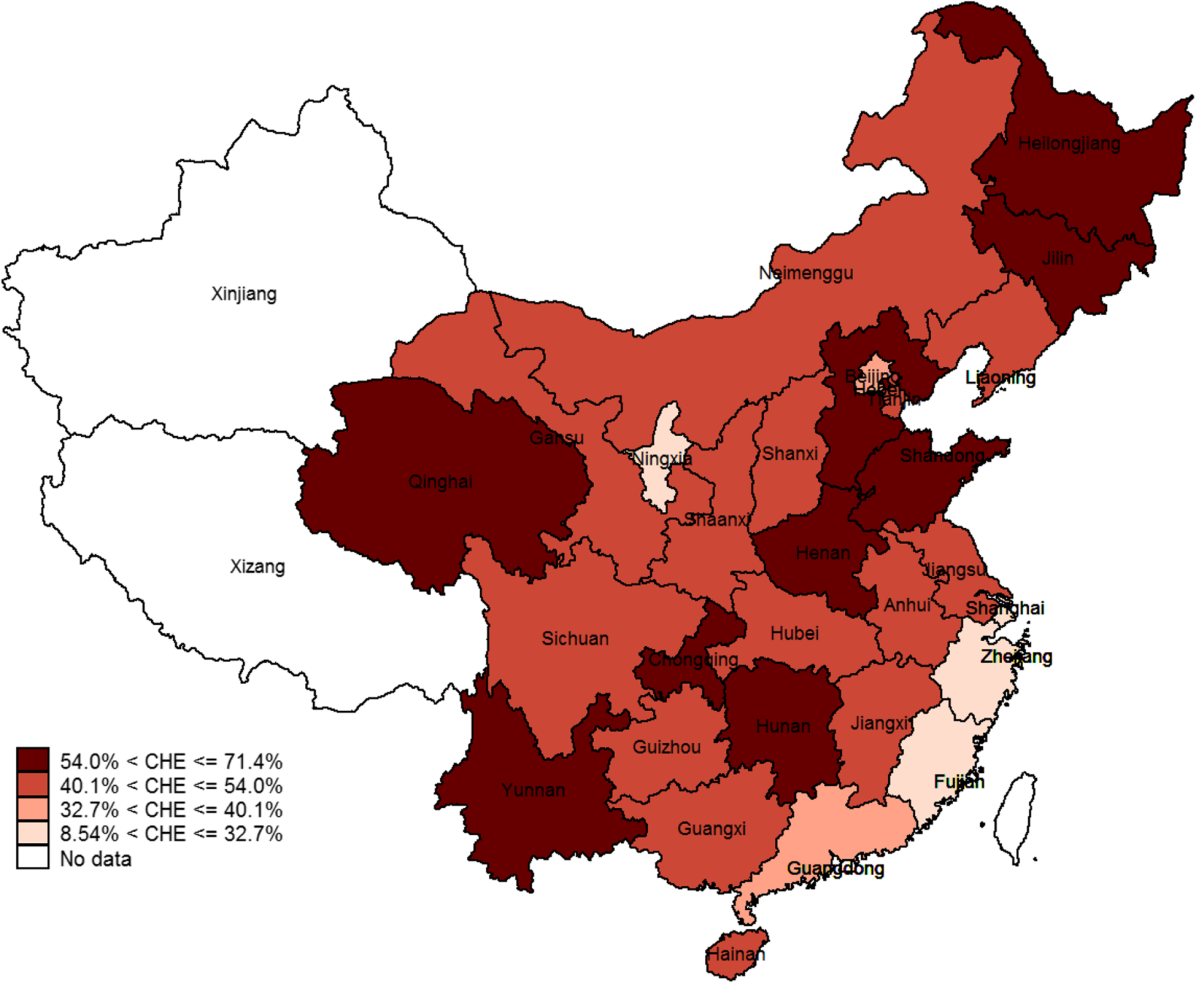
Percentage of low-income households with CHE across provinces in China

Figure 4 shows CHE across China’s provinces in 2014. Heilongjiang, Jilin, Hebei, Henan, Shandong, Chongqing, Hunan, Yunnan, and Qinghai had the highest percentages of low-income households with CHE at over 54.0%.

**Fig. 5:**
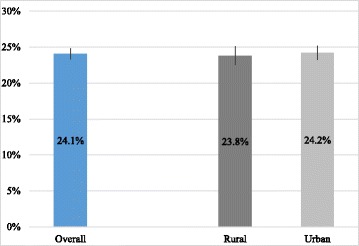
Percentage of low-income households receiving MFA cash aid in rural and urban China

Figure 5 shows the data relating to MFA cash aid: 24.1% of low-income households received cash aid, with no significant difference between rural (23.8%) and urban (24.2%) areas.

Table 4 presents the associations between between MFA cash aid and CHE, and household SHI enrollment and CHE. Both models fit the data well, with Wald chi2 equal to 787.51 (*p* < 0.001) for the MFA cash aid model and 3402.95 (Prob > chi2 = 0.000) for the SHI enrollment model.

**Table 4:** Results of multilevel logistic analysis: MFA cash aid and CHE using the Q2 sample; SHI enrollment and CHE using the Q3 sample

CHE (1 = with CHE; 0 = no CHE)	Q2 sample (*N* = 1490; Province Group = 28; County Group = 135)		Q3 sample (*N* = 10,344; Province Group = 29; County Group = 158)	
	OR	95% CI	OR	95% CI
Household SHI enrollment				
No participation	-		reference	
Partial participation	-		0.843	(0.693, 1.025)
Full participation	-		0.760**	(0.626, 0.924)
MFA cash aid	1.009	(0.744, 1.367)	-	
Log (total medical cost)	2.774***	(2.364, 3.256)	2.172***	(2.026, 2.328)
Urban	0.723	(0.449, 1.163)	0.620***	(0.474, 0.811)
Number of males	0.942	(0.808, 1.099)	0.969	(0.910, 1.031)
Number of older adults	0.896	(0.737, 1.088)	1.173***	(1.077, 1.276)
Number of children	0.655**	(0.484, 0.886)	0.806***	(0.718, 0.905)
Adjusted household health score	1.217**	(1.065, 1.392)	1.358***	(1.310, 1.407)
Number of people with chronic illnesses	0.827*	(0.692, 0.988)	0.864**	(0.791, 0.945)
Number of people with serious illnesses	1.201	(0.909, 1.587)	0.925	(0.813, 1.053)
Number of people needing long-term care	0.916	(0.741, 1.133)	0.989	(0.924, 1.060)
Number of people with high school education and above	0.725**	(0.598, 0.878)	0.660***	(0.610, 0.714)
Number of unemployed	1.253**	(1.070, 1.467)	1.291***	(1.184, 1.406)
Log (per capita household income)	0.984	(0.966, 1.003)	0.986**	(0.977, 0.996)

*OR* odds ratio, *CI* confidence interval, *SHI* social health insurance, *MFA* Medical Financial Assistance, *CHE* catastrophic health expenditure; * = *p* < 0.05; ** = *p* < 0.01; *** = *p* < 0.001

We detected no significant association between MFA cash aid and CHE (OR = 1.009; 95% CI = 0.744–1.367). Among the covariates, higher total medical costs, worse health status, and the number of unemployed household members were significantly associated with a higher likelihood of CHE. Having more children, more members with chronic conditions, and more members with a high school education and above were inversely associated with the likelihood of CHE. Comparable results were obtained when testing the sensitivity of the results using the propensity score matching model, where the association between MFA cash aid and CHE was also statistically insignificant (see Additional file 1: Table S5). Additional file 1: Table S4 shows that no significant association was found between MFA cash aid and CHE in either rural or urban areas. Additional file 1: Table S7 shows no significant association between MFA cash aid and CHE in any of the four regions.

Regarding the association between SHI enrollment and CHE, when controlling for total medical costs and other covariates, full SHI enrollment was significantly associated with lower likelihood of CHE (OR = 0.760, 95% CI = 0.626–0.924). A household with all members enrolled in SHI programs was 24.0% less likely to have CHE. Among other covariates, the likelihood of CHE increased with total medical cost, the number of older adults, worse health status, and the number of unemployed members; and decreased with living in urban areas, the number of children, the number of members with chronic illnesses, the number of members with high school education and above, and higher household income. Additional file 1: Table S4 shows that SHI enrollment had no significant association with CHE in rural areas, but had a significantly negative association with CHE in urban areas. Additional file 1: Table S7 shows that full SHI enrollment had a significantly negative association with CHE in eastern and central regions.

## Discussion

Using data from a 2014 nationally representative database, this study found that about one quarter of low-income households were not enrolled in SHI programs and nearly half of those households did not receive MFA subventions for SHI enrollment. In addition, over half of the low-income households suffered from catastrophic health spending, while only a quarter of them received MFA cash aid. A three-level logistic analysis revealed that MFA subvention had no significant association with SHI enrollment in low-income households, that MFA cash aid had no significant association with the CHE status of low-income households, and that household SHI enrollment played an important role in reducing households’ catastrophic health spending.

The findings reveal the limited role of the MFA subsidy in encouraging low-income households to enroll in SHI programs and mitigating the financial risks of illness. The findings agree with previous studies that have found no significant association between a special MFA benefit package and the presence of substantial medical debt in rural areas of Chongqing Municipality [39], low coverage of enrollment and benefit levels of the MFA in four counties in Hubei Province and Sichuan Province [42], and a minor relationship between MFA cash aid and the financial burden of urban low-income households in the three counties of Hebei Province, Hubei Province, and Chongqing Municipality [48].

The non-significant association between MFA subvention and SHI enrollment could be due to limited eligibility and funding for a MFA subvention. As Additional file 1: Table S1 shows, in many localities, a MFA subvention for SHI enrollment is only provided to the extremely poor. Even if a poor household receives a MFA subvention for SHI enrollment, it may choose not to participate in SHI programs if the subvention funds cannot cover the total cost of the SHI premium. In addition, the MFA subvention for SHI enrollment is provided to households in cash, which allows them to use it for other purposes, such as buying food. This may be a key reason that a quarter of low-income households were not enrolled in SHI programs.

We have observed no significant association between MFA cash aid and CHE. There are two plausible explanations. First, the cash aid is mainly funded by local government and its threshold and ceiling of reimbursement are directly impacted by the local governments’ fiscal capacity. If a local government has a restricted budget, the MFA cash aid may have high thresholds and low ceilings for reimbursement. This may also explain why only a quarter of low-income households received MFA cash aid. Taking Shandong Province as an example, the threshold for MFA cash aid was USD163 for outpatient services (see Additional file 1: Table S1). The average cost of outpatient services was USD35 per visit in 2014, suggesting a large gap between the threshold of the MFA and the average cost of medical services [59]. Second, in many areas, MFA cash aid covers the cost of limited kinds of serious illnesses, as shown in Additional file 1: Table S1.

This study advocates the establishment of a “pro-poor” medical financial assistance policy in China that can respond to the international trend of investing in health as a major avenue towards poverty alleviation [60]. China has made remarkable progress in enacting and legislating the MFA program, extending coverage, and improving funding management [40, 41]. Further efforts should be devoted to at least two aspects: (1) ensuring low-income households’ enrollment in SHI programs. In this study, SHI has been found to be an efficient institution for mitigating catastrophe. The government may consider free SHI enrollment for all types of low-income households, rather than just providing subsidies for enrollment; (2) increasing the benefit package of MFA cash aid by reducing its threshold and expanding coverage to more serious diseases. At present, it is too small to alleviate catastrophic health spending in low-income households. Increasing funds for MFA cash aid could construct an effective safety net for the poor.

Our study has the following limitations. Firstly, the data in this study was extracted from one cross-sectional survey, with the result that is not possible to assess causal relationships. Secondly, the health spending data used were collected through a self-reported survey, that may not accurately reflect medical costs.

As the MFA, together with SHI programs, will continue to be major measures for financial risk protection in low-income households, future studies should focus on examining the causal effects of various policy components of MFA subvention and cash aid, respectively on SHI enrollment and financial risk protection using longitudinal data. For example, this study identified no significant association between MFA cash aid and CHE. Could this lack of a significant association be caused by high thresholds, low cash aid, or low ceilings for the cash aid? Future studies on this question may enable policy makers to make accurate adjustments to MFA benefit packages.

## Conclusions

This study suggests that the MFA is not currently an effective supplement in terms of either subsidizing SHI enrollment or preventing financial risks through providing cash aid. As a result, it is neither a useful buffer in the cycle between poverty and poor health, nor a “pro-poor” health policy in the model advocated by the World Health Organization [18]. Compared with the poor performance of the MFA, SHI programs, with nearly nationwide coverage, play an important role in alleviating the health spending-related impoverishment experienced by low-income households.

## Additional file

Additional file 1:
**Table S1.** Eligibility and benefit package of the Medical Financial Assistance program across provinces in China. **Supplementary Text. Fig. S1** Percentage of low-income households having the CHE, by urban/rural, MFA cash aid status and SHI status. **Table S2** Summary of variables used in the three multilevel logistical models in rural and urban areas. **Table S3** Results of multilevel logistic analysis in rural and urban areas: MFA subvention and SHI enrollment using the Q1 sample. **Table S4** Results of multilevel logistic analysis by rural and urban areas: MFA cash aid and the CHE; and SHI enrollment and the CHE. **Table S5** Results of propensity score matching analysis using the Q2 sample: Average treatment effects on the treated (ATT) for treatment of CHE. **Table S6** Summary of variables used in multilevel logistic models including the interaction between main independent variables and regions. **Table S7** Results of multilevel logistic models including the interaction between main independent variables and regions. (DOCX 64 kb)

## Abbreviations

CHE: Catastrophic health expenditure
CI: Confidence Interval
ESPSS: Evaluating social policy supporting system for vulnerable families in Urban and Rural China
MFA: Medical Financial Assistance
N: Sample size
OPE: Out-of-pocket health expenditure
OR: Odds ratio
SHI: Social Health Insurance
WHO: World Health Organization
