# Supplementary material for: Is the medical financial assistance program an effective supplement to social health insurance for low-income households in China? A cross-sectional study
Source: Int J Equity Health. 2017 Aug 1;16:138. doi: 10.1186/s12939-017-0638-3 (PMC5540305; doi:10.1186/s12939-017-0638-3)
Supplement: Additional file 1: — Table S1. Eligibility and benefit package of the Medical Financial Assistance program across provinces in China. Supplementary Text. Fig. S1 Percentage of low-income households having the CHE, by urban/rural, MFA cash aid status and SHI status. Table S2 Summary of variables used in the three multilevel logistical models in rural and urban areas. Table S3 Results of multilevel logistic analysis in rural and urban areas: MFA subvention and SHI enrollment using the Q1 sample. Table S4 Results of multilevel logistic analysis by rural and urban areas: MFA cash aid and the CHE; and SHI enrollment and the CHE. Table S5 Results of propensity score matching analysis using the Q2 sample: Average treatment effects on the treated (ATT) for treatment of CHE. Table S6 Summary of variables used in multilevel logistic models including the interaction between main independent variables and regions. Table S7 Results of multilevel logistic models including the interaction between main independent variables and regions. (DOCX 64 kb) [file 12939_2017_638_MOESM1_ESM.docx]

**Additional file 1**

Table S1. Eligibility and benefit package of the Medical Financial Assistance program across provinces in China

| Provinces | Eligibility | Subvention for SHI enrolment | Services covered | Serious illnesses covered | Thresholds ($) | Cash aid rates (% of OPE) | Ceilings ($) | Year of establishment |
| --- | --- | --- | --- | --- | --- | --- | --- | --- |
| 1.Beijing | MLSS; EPR; LIF | Fully funded | OP; IP; Maternity Services; Surgery; Medical tests; Bed fee of IP | 15 kinds | 81(before 2014) | EPR: 100%; OP & IP: 70%; Serious illnesses: 75%; Surgery & medical tests: 20%; Bed fee: 50% | OP: 651; IP: 6,512; Serious illnesses: 13,023; Maternity services:1,302 | 2014 |
| 2.Tianjing | MLSS; EPR; LIF(2014) | Fully funded | OP; IP; Surgery; Medical tests | N/A | N/A | IP: sectional rates (60% if >3,256; 80% if <3,256 in 2014)  Surgery & medical tests:10% | OP (EPR): 33; OP (MLSS): 10; IP: 16,279 | 2009 |
| 3.Hebei | MLSS; EPR; LIF | 60%-100% | OP; IP | 7 kinds | LIF: 3,256 | OP (EPR): 100%; IP (EPR): 80%; IP (MLSS): sectional rates (70% if <326; 50% if >814); IP (LIF): 20% | OP (EPR): 33; IP (EPR): 977; IP (MLSS): 977; IP (LIF): 977 | 2010 |
| 4.Inner Mongolia | MLSS; EPR; LIF | 50% | OP; IP | 21 kinds | LIF: 4,884 for total medical costs with OPE over 814 | IP: sectional rates (100% if <163; 70% if >163); IP on serious illnesses: 80%; LIF:60% | OP: 488; IP: 326; IP on serious illnesses: 9,768; IP (LIF): 260 | 2013 |
| 5.Xinjiang | MLSS; EPR | EPR: fully funded | OP; IP | N/A | General illnesses: 16-49; Serious illnesses: 163-326 | IP (EPR): 100%; IP (MLSS): sectional rates (50% if <814; 85% if >3,256) | OP (EPR): 33; IP (MLSS): 4,884 | 2010 |
| 6.Qinhai | MLSS;EPR | Fully funded | OP; IP; Medical tests | N/A | LIF: 814; Other residents: 16,279 | Medical tests: 10%; IP: 85%-90%; IP (LIF): 50%; Other residents: 20%-40% | OP: 20-81; IP: 4,884; IP (LIF): 1,627; Other residents: 32,558 | 2010 |
| 7.Gansu | MLSS; EPR | MLSS & EPR: fully funded | OP; IP; Maternity services | N/A | N/A | IP (EPR):100%; IP (MLSS): 70% | OP (EPR): 33; OP (MLSS): 3; IP: 6,512; IP on serious illnesses: 13,023; Maternity Services (MLSS): 33-65 | 2014 |
| 8.Ninxia | MLSS; EPR | Fully funded | OP; IP | N/A | MLSS & EPR: 4,884 for serious illnesses | OP (EPR): 90%; OP (MLSS): 50%; IP (EPR): 90%; IP (MLSS): 70%; Serious illnesses: 50%-60% | OP (EPR): 488; OP (MLSS): 326; IP (4,884); Serious illnesses: 13,023 | 2015 |
| 9.Sichuan | MLSS | N/A | OP; IP; Maternity services | N/A | N/A | IP-MLSS: sectional rates (60% for tertiary Grade A hospitals) | OP (MLSS): 3-16; IP (MLSS): 814-1,627; Maternity Services (MLSS): 81; Serious illnesses: 163 | 2015 |
| 10.Chongqing | MLSS; EPR; LIF; Poor college students | EPR: fully funded;  Others: 8 | OP; IP | 22 kinds | One-time IP (not on the list of 22 kinds of serious illnesses): 4,884 | OP (MLSS): 60%; IP (MLSS & EPR): 60%; IP (LIF): 40%; IP on serious illnesses (MLSS & EPR): 70%; IP on serious illnesses (LIF): 50% | OP-EPR:>33  OP (MLSS): >16; IP: >977; IP on serious illnesses: 16,279; One-time IP: 9,768 | 2012 |
| 11.Shaanxi | MLSS; EPR; LIF; Entitled servicemen subject to special care | EPR: fully funded | OP; IP | 7 kinds | Serious illnesses: 3,256 | OP (EPR): 100%; OP (MLSS): (OPE - 3*MLSS line) * 65%; IP (EPR): 100%; IP (MLSS & entitled servicemen): 65%; IP (LIF): (OPE – average income)*65% | OP: 3,256; IP: 3,256; IP on serious illnesses: 16,279 | 2014 |
| 12.Shanxi | MLSS; EPR; LIF | EPR: fully funded; Others: 50% | OP; IP | N/A | Serious illnesses: 3,256 | IP (EPR): 100%; IP (MLSS): 60% | N/A | 2013 |
| 13.Henan | MLSS; EPR | N/A | OP; IP | N/A | N/A | OP: 50%; IP: 35%-50% | OP: 814; IP: 1,627; IP on serious illnesses: 3,256 | 2013 |
| 14.Anhui | MLSS; EPR; LIF | MLSS & EPR: fully funded; LIF: 50% | OP; IP | 14 kinds | N/A | IP (MLSS & EPR): 65%; IP (LIF): 35% | OP (EPR): 81; IP (MLSS): 3,256; IP (EPR): 4,884; IP (LIF): 2,442 | 2014 |
| 15.Jiangxi | MLSS; EPR; LIF | MLSS & EPR: fully funded | OP; IP | N/A | N/A | OP (EPR):100%; OP (MLSS): 50%-60%; IP (EPRS): 100%; IP (MLSS): 70%; IP on serious illnesses (MLSS): 70%; IP on serious illnesses (LIF): 50% | OP (MLSS): 163; OP on serious illnesses (MLSS): 1,627-3,256; IP (MLSS): 4,884; IP on serious illnesses (MLSS): 8,140 | 2015 |
| 16.Shandong | MLSS; EPR | 50%-100% | OP; IP | 5 kinds | OP:163; Serious illnesses: 4,884 | OP: 20%; IP: 50%; Serious illnesses: 20%-30% (accumulated with IP) | OP: 326; IP: 1,627; Serious illnesses: 8,140 (accumulated with IP) | 2011 |
| 17.Guangdong | MLSS; EPR; LIF; Entitled servicemen; Poor college students | Fully funded | OP; IP | N/A | N/A | OP (MLSS & EPR): 100%; IP (MLSS & EPR): 100%; IP (LIF): sectional rates (90% if <8,140; 80% if >8,140) | OP (MLSS & EPR): 195; IP (MLSS & EPR): 18,140 | 2016 |
| 18.Fujian | MLSS; EPR; LIF | MLSS & EPR: fully funded | OP; IP; Maternity services | N/A | N/A | IP (MLSS & EPR): 50%; IP (LIF): 30% | MLSS & EPR: 1,627; LIF: 488 | 2009 |
| 19.Guangxi | MLSS; EPR; LIF | EPR: fully funded | OP; IP | N/A | Serious illnesses: 4,884 | OP: 60%-70%; IP: 50% | OP:130-163; IP: 1,627-2,440; IP on serious illnesses: 6,512 | 2011 |
| 20.Yunnan | MLSS; EPR; LIF | Partial funding | OP; IP | 22 kinds | LIF: 814 | OP (EPR): 60%-100%; IP (EPR): 100%; IP (MLSS): 40%; IP (LIF): 30%; IP on serious illnesses: 50%-100% | OP (EPR): 244; IP: 2,442; IP on serious illnesses: 8,140 | 2015 |
| 21.Hainan | MLSS; EPR; LIF | N/A | OP; IP | 7 kinds | LIF: 814 | EPR: 100%; MLSS: 60%; LIF: 50% | 488 | 2010 |
| 22.Liaoning | MLSS; EPR; LIF | MLSS & EPR: fully funded | OP; IP | 8 kinds | N/A | IP (MLSS & EPR): 70%; Serious illnesses:50% | IP (MLSS & EPR): 814; Serious illnesses:1,627 | 2014 |
| 23.Jilin | MLSS; EPR | EPR: fully funded | OP; IP | N/A | MLSS: 15% of ceilings for Serious Illnesses Insurance; OP on serious illnesses: 65 | IP (EPR): 100%; IP (MLSS): 70%; OP on serious illnesses: 30% | EPR: 3,256; MLSS: 1,627 | 2015 |
| 24.Heilongjiang | MLSS; EPR | EPR: fully funded | OP; IP | 16 kinds | N/A | OP (EPR): 100%; IP (EPR): 80%; IP (MLSS): 40%; IP on serious illnesses: 50%-90% | OP (EPR): 33; IP (EPR): 1,302; IP (MLSS): 488; IP on serious illnesses: 977-1,627 | 2009 |
| 25.Zhejiang | MLSS; EPR; LIF | N/A | OP; IP | N/A | LIF: MLSS line | EPR: 100%; MLSS: 70%; LIF: 60% | 13,023 | 2014 |
| 26.Jiangsu | MLSS; EPR; LIF; Entitled servicemen | EPR: fully funded | OP; IP | N/A | LIF: 1,627 | MLSS & EPR: 85%; LIF: 50% | OP (MLSS & EPR): 326; LIF: 1,627 | 2016 |
| 27.Shanghai | MLSS; EPR; LIF | N/A | OP; IP | N/A | N/A | EPR: 100%; MLSS: 80%; LIF: 70% | 13,023 | 2015 |
| 28.Hunan | MLSS; EPR; LIF | EPR: fully funded | OP; IP | 17 kinds | LIF: 488; Serious illnesses: 4,884 | OP (EPR): 100%; OP (MLSS ): 100%; IP (EPR): 100%; IP (MLSS): 60%-70%; IP (LIF): 50% | OP:163-195; IP: 1,627; IP on serious illnesses: 8,140 | 2015 |
| 29.Hubei | MLSS; EPR; LIF | MLSS & EPR: fully funded | OP; IP | 21 kinds | 3,256; LIF: 4,884 for serious illnesses | IP:70%; IP (LIF): 60%; IP on serious illnesses: 70%; IP on serious illnesses (LIF): 60% | 977; IP on serious illnesses: 3,256; IP on serious illnesses (LIF): 1,627 | 2013 |
| 30.Guizhou | MLSS; EPR; LIF; Entitled servicemen | EPR: fully funded | OP; IP | N/A | N/A | IP: 60% | IP: 1,627 | 2013 |

Sources: see references. Notes: MLSS=households enrolled on the Minimum Living Standard Scheme; EPR=extremely poor residents, including “Sanwu”, urban residents with no income, labor capacity, or caregivers; “Wubao”, rural residents with no income, labor capacity, or caregivers; “Tekun”, households defined as extremely poor by the Draft Decree on Social Assistance; LIF=low-income families not enrolled on the MLSS that was identified by local governments, with the criterion that a monthly family income was between 100% and 120%–150% of the local MLSS line; OP=Outpatient services; IP=Inpatient services. Tibet was not included because of its free medical care scheme for all residents.

Supplementary Text

Strategies of selecting eligible low-income households for MFA cash aid and generating the Q2 sample

The analysis on the association between MFA cash aid and CHE may encounter the problem of self-selection bias due to the policy design of thresholds for MFA cash aid. Households not receiving MFA cash aid may be healthier than those receiving it, and therefore present OPE below the threshold, ineligible for MFA cash aid. As a result, receiving MFA cash aid *per se* denotes high OPE and the probability of CHE. However, MFA cash aid is not a mandatory but a voluntary compensation. Households whose OPE exceeds the threshold for MFA cash aid must approach local county-level Bureaus of Civil Affairs in order to apply for MFA cash aid. Because of information asymmetry and distance problems, some eligible households may not apply, even if their OPE has exceeded the threshold. The presence of these eligible-but-no-cash-aid households provided the basis for solving the problem of self-selection bias associated with MFA cash aid because their OPE should present no systematic difference from the pre-MFA cash aid OPE (current OPE+MFA cash aid received) of those who have received it. Households of both types are eligible to apply for MFA cash aid.

To identify these eligible households, we used a resampling method, with the OPE spent on serious illnesses as the target. We used the OPE spent on serious illnesses rather than the general OPE of all household members because the latter may include individual health spending that is too small to be eligible for MFA cash aid. Among all the medical needs of low-income households, serious illness is the most salient factor inducing CHE. The MFA program across counties generally identifies a fixed list of serious illnesses and establishes a special cash aid and fixed thresholds. If a household spent a certain sum of OPE on serious illnesses and did not apply for MFA cash aid, we deemed it to be an eligible group that had given up cash aid.

We used the average value of the OPE plus MFA cash aid spent on serious illnesses (pre-MFA cash aid OPE on serious illnesses) among households that received MFA cash aid in each county as the cut-off point, because the threshold is county-specific. To insure normal distribution of pre-MFA cash aid OPE on serious illnesses, we used T-scores (calculated from Z-scores, mean=50, standard deviation=10, T-score=50-10*Z-score) of pre-MFA cash aid OPE to facilitate selection. First, we generated by-county mean (*mean_t*) and maximum values (*max_t*) of T-scores among households receiving MFA cash aid. Second, in each county, we identified households that did not receive MFA cash aid but had T-scores higher than *mean_t* and lower than *max_t*. We defined these households as Group 1 (eligible-but-no-cash-aid households). Third, we selected households receiving MFA cash aid and with T-scores higher than *mean_t* and lower than *max_t*. We defined these as Group 2 (eligible-and-receiving-cash-aid households). Fourth, we combined the two groups to form a new sample (the Q2 sample). After resampling, the mean value of pre-MFA cash aid OPE on serious illnesses was $2,150 (95% CI: 1,995.77–2,305.16) for Group 1 and $2,414 (95% CI: 2,136.91–2,691.51) for Group 2, indicating that there was no systematic difference between the two groups in terms of pre-MFA cash aid OPE on serious illnesses, thus avoiding self-selection bias.

Figure S1. Percentage of low-income households having the CHE, by urban/rural, MFA cash aid status and SHI status.

Table S2. Summary of variables used in the three multilevel logistical models in rural and urban areas

|  | Q1 sample | | | Q2 sample | | | Q3 sample | | |
| --- | --- | --- | --- | --- | --- | --- | --- | --- | --- |
|  | N | Mean | SD | N | Mean | SD | N | Mean | SD |
| **Rural** | | | | | | | | | |
| CHE | 3,706 | 0.60 | 0.49 | 559 | 0.75 | 0.43 | 4,188 | 0.59 | 0.49 |
| Household SHI enrollment |  |  |  |  |  |  |  |  |  |
| No participation | 3,742 | 0.25 | 0.43 | - | - | - | 4,228 | 0.22 | 0.41 |
| Partial participation | - | - | - | - | - | - | 4,228 | 0.11 | 0.32 |
| Full participation | 3,742 | 0.75 | 0.43 | - | - | - | 4,228 | 0.67 | 0.47 |
| MFA cash aid | 3,743 | 0.24 | 0.42 | 556 | 0.48 | 0.50 | - | - | - |
| MFA subvention for SHI enrollment |  |  |  |  |  |  |  |  |  |
| No subvention (reference) | 3,562 | 0.55 | 0.50 | - | - | - | - | - | - |
| Partial subvention | 3,562 | 0.21 | 0.41 | - | - | - | - | - | - |
| Full subvention | 3,562 | 0.24 | 0.43 | - | - | - | - | - | - |
| Log (total medical cost) | 3,375 | 7.01 | 1.71 | 562 | 8.18 | 1.09 | 3,827 | 7.04 | 1.70 |
| Number of males | 3,746 | 1.58 | 0.96 | 562 | 1.78 | 1.05 | 4,232 | 1.64 | 0.97 |
| Number of older adults | 3,746 | 0.68 | 0.79 | 562 | 0.74 | 0.88 | 4,232 | 0.67 | 0.78 |
| Number of children | 3,746 | 0.10 | 0.35 | 562 | 0.12 | 0.38 | 4,232 | 0.12 | 0.37 |
| Adjusted household health score | 3,741 | 3.78 | 2.11 | 561 | 3.82 | 1.89 | 4,232 | 3.39 | 0.90 |
| Number of people with chronic illnesses | 3,742 | 0.77 | 0.82 | 561 | 0.75 | 0.88 | 4,228 | 0.78 | 0.83 |
| Number of people with serious illnesses | 3,744 | 0.31 | 0.54 | 562 | 1.14 | 0.36 | 4,229 | 0.32 | 0.54 |
| Number of people needing long-term care | 3,745 | 0.46 | 0.69 | 562 | 0.71 | 0.75 | 4,231 | 0.47 | 0.69 |
| Number of people with high school education and above | 3,746 | 0.28 | 0.62 | 562 | 0.34 | 0.68 | 4,232 | 0.31 | 0.65 |
| Number of unemployed | 3,746 | 1.05 | 0.86 | 562 | 1.33 | 0.86 | 4,232 | 1.06 | 0.86 |
| Log (per capita household income) | 3,746 | 3.78 | 8.09 | 562 | 4.02 | 7.66 | 4,232 | 3.88 | 7.94 |
| **Urban** | | | | | | | | | |
| CHE | 5,683 | 0.44 | 0.50 | 943 | 0.65 | 0.48 | 7,198 | 0.43 | 0.50 |
| Household SHI enrollment |  |  |  |  |  |  |  |  |  |
| No participation | 5,779 | 0.31 | 0.46 | - | - | - | 7,326 | 0.24 | 0.43 |
| Partial participation | - | - | - | - | - | - | 7,326 | 0.21 | 0.41 |
| Full participation | 5,779 | 0.69 | 0.46 | - | - | - | 7,326 | 0.55 | 0.50 |
| MFA cash aid | 5,782 | 0.23 | 0.42 | 953 | 0.42 | 0.49 |  |  |  |
| MFA subvention for SHI enrollment |  |  |  |  |  |  |  |  |  |
| No subvention (reference) | 4,965 | 0.46 | 0.50 | - | - | - | - | - | - |
| Partial subvention | 4,965 | 0.28 | 0.45 | - | - | - | - | - | - |
| Full subvention | 4,965 | 0.26 | 0.44 | - | - | - | - | - | - |
| Log (total medical cost) | 5,161 | 7.04 | 1.65 | 959 | 8.19 | 1.08 | 6,615 | 7.11 | 1.66 |
| Number of males | 5,791 | 1.39 | 0.87 | 959 | 1.56 | 0.87 | 7,338 | 1.46 | 0.89 |
| Number of older adults | 5,791 | 0.49 | 0.72 | 959 | 0.60 | 0.78 | 7,338 | 0.50 | 0.73 |
| Number of children | 5,791 | 0.07 | 0.28 | 959 | 0.09 | 0.31 | 7,338 | 0.08 | 0.29 |
| Adjusted household health score | 5,779 | 3.79 | 2.03 | 958 | 3.98 | 1.95 | 7,338 | 3.26 | 0.92 |
| Number of people with chronic illnesses | 5,783 | 0.76 | 0.81 | 958 | 0.79 | 0.86 | 7,329 | 0.79 | 0.83 |
| Number of people with serious illness | 5,786 | 0.27 | 0.51 | 959 | 1.14 | 0.39 | 7,333 | 0.29 | 0.53 |
| Number of people needing long-term care | 5,790 | 0.39 | 0.61 | 959 | 0.69 | 0.68 | 7,337 | 0.42 | 0.63 |
| Number of people with high school education and above | 5,791 | 0.67 | 0.91 | 959 | 0.81 | 0.95 | 7,338 | 0.76 | 0.96 |
| Number of unemployed | 5,791 | 0.61 | 0.73 | 959 | 0.84 | 0.79 | 7,338 | 0.65 | 0.75 |
| Log (per capita household income) | 5,791 | 4.32 | 8.41 | 959 | 4.30 | 8.48 | 7,338 | 4.52 | 8.16 |

Table S3. Results of multilevel logistic analysis in rural and urban areas: MFA subvention and SHI enrollment using the Q1 sample

| Household SHI enrollment (1=all enrolled; 0= none enrolled) | Rural (N=3,554) | Urban (N=4,944) |  |
| --- | --- | --- | --- |
|  | OR (95% CI) | OR (95% CI) | |
| MFA subvention for SHI enrollment |  |  | |
| No subvention (reference) | reference | reference | |
| Partial subvention | 1.226 (0.694, 2.166) | 0.954 (0.673, 1.353) | |
| Full subvention | 1.493 (1.023, 2.179)* | 1.029 (0.770, 1.374) | |
| Number of males | 0.972 (0.778, 1.213) | 0.950 (0.835, 1.082) | |
| Number of older adults | 1.051 (0.843, 1.309) | 1.070 (0.911, 1.256) | |
| Number of children | 1.065 (0.689, 1.648) | 0.946 (0.694, 1.288) | |
| Adjusted household health score | 0.992 (0.934, 1.054) | 1.016 (0.954, 1.083) | |
| Number of people with chronic illnesses | 1.444 (1.188, 1.755)*** | 1.490 (1.235, 1.797)*** | |
| Number of people with serious illness | 1.223 (1.000, 1.496)* | 1.229 (0.956, 1.580) | |
| Number of people needing long-term care | 1.021 (0.864, 1.206) | 0.959 (0.763, 1.204) | |
| Number of people with high school education and above | 1.068 (0.902, 1.264) | 1.098 (0.975, 1.236) | |
| Number of unemployed | 1.029 (0.845, 1.253) | 0.806 (0.650, 1.000)* | |
| Log (per capita household income) | 1.024 (1.010, 1.038)*** | 1.028 (1.013, 1.042)*** | |

Notes: *=p<0.05, **=p<0.01, ***=p<0.001.

Table S4. Results of multilevel logistic analysis by rural and urban areas: MFA cash aid and the CHE; and SHI enrollment and the CHE

| CHE (1=with CHE; 0=no CHE) | Q2 sample | | Q3 sample | |
| --- | --- | --- | --- | --- |
|  | OR (95% CI)  (Rural, N=552) | OR (95% CI)  (Urban, N=938) | OR (95% CI)  (Rural, N=3,802) | OR (95% CI)  (Urban, N=6,542) |
| Household SHI enrollment |  |  |  |  |
| No participation | - | - | reference | reference |
| Partial participation | - | - | 1.009 (0.707, 1.439) | 0.759 (0.597, 0.965)* |
| Full participation | - | - | 0.862 (0.701, 1.059) | 0.709 (0.546, 0.922)* |
| MFA cash aid | 0.942 (0.514, 1.725) | 1.044 (0.650, 1.678) | - | - |
| Log (total medical cost) | 3.123 (2.298, 4.242)*** | 2.621 (2.163, 3.177)*** | 1.898 (1.730, 2.083)*** | 2.413 (2.232, 2.609)*** |
| Number of males | 0.867 (0.670, 1.120) | 0.938 (0.757, 1.162) | 0.913 (0.829, 1.006) | 0.998 (0.915, 1.089) |
| Number of older adults | 0.853 (0.623, 1.168) | 0.912 (0.735, 1.131) | 1.028 (0.914, 1.157) | 1.236 (1.126, 1.356)*** |
| Number of children | 0.514 (0.273, 0.967)* | 0.816 (0.565, 1.179) | 0.870 (0.731, 1.035) | 0.779 (0.671, 0.905)** |
| Adjusted household health score | 1.109 (0.902, 1.363) | 1.281 (1.123, 1.460)*** | 1.315 (1.225, 1.413)*** | 1.374 (1.319, 1.432)*** |
| Number of people with chronic illnesses | 0.937 (0.687, 1.279) | 0.781 (0.632, 0.966)* | 1.006 (0.869, 1.163) | 0.798 (0.729, 0.874)*** |
| Number of people with serious illness | 1.199 (0.441, 3.259) | 1.264 (0.829, 1.928) | 0.915 (0.757, 1.106) | 0.910 (0.797, 1.039) |
| Number of people needing long-term care | 0.839 (0.538, 1.307) | 0.998 (0.761, 1.308) | 0.872 (0.780, 0.974)* | 1.069 (0.967, 1.181) |
| Number of people high school education and above | 0.508 (0.348, 0.739)*** | 0.792 (0.666, 0.941)** | 0.539 (0.457, 0.635)*** | 0.698 (0.645, 0.755)*** |
| Number of unemployed | 1.284 (0.991, 1.664) | 1.322 (0.990, 1.767) | 1.389 (1.229, 1.570)*** | 1.271 (1.152, 1.401)*** |
| Log (per capita household income) | 1.011 (0.982, 1.042) | 0.972 (0.949, 0.995)* | 0.987 (0.977, 0.996)** | 0.985 (0.974, 0.997)* |

Notes: *=p<0.05, **=p<0.01, ***=p<0.001.

Table S5. Results of propensity score matching analysis using the Q2 sample: Average treatment effects on the treated (ATT) for treatment of CHE

| Matching method | Number in the treated group | Number in the controlled group | ATT | SE | Z | P |
| --- | --- | --- | --- | --- | --- | --- |
| K-nearest neighbor matching(k=4) | 656 | 826 | -0.005 | 0.029 | -0.20 | 0.842 |
| One-to-one matching | 656 | 826 | -0.006 | 0.036 | -0.17 | 0.865 |
| Radius matching | 656 | 825 | -0.011 | 0.023 | -0.47 | 0.638 |
| Kernel matching | 656 | 826 | -0.002 | 0.023 | -0.10 | 0.922 |

Notes: The PSM analysis was conducted to solve the endogeneity problem in the relationship between MFA cash aid and CHE and to test the robustness of the multilevel model. As both the MFA subvention and SHI enrollment were not binary variable, we did not use the PSM to test the associations between MFA subvention and SHI enrollment and between SHI enrollment and CHE. The term “treated” in the table refers to MFA cash aid.

Table S6. Summary of variables used in multilevel logistic models including the interaction between main independent variables and regions

|  | Q1 sample | | | | Q2 sample | | | | Q3 sample | | | |
| --- | --- | --- | --- | --- | --- | --- | --- | --- | --- | --- | --- | --- |
|  | | N | Mean | SD | | N | Mean | SD | | N | Mean | SD |
| CHE | | 9,389 | 0.50 | 0.50 | | 1,502 | 0.69 | 0.46 | | 11,386 | 0.49 | 0.50 |
| Household SHI enrollment | |  |  |  | |  |  |  | |  |  |  |
| No SHI enrollment | | 9,521 | 0.28 | 0.45 | | - | - | - | | 11,554 | 0.23 | 0.42 |
| Partial SHI enrollment | | - | - | - | | - | - | - | | 11,554 | 0.18 | 0.38 |
| Full SHI enrollment | | 9,521 | 0.72 | 0.45 | | - | - | - | | 11,554 | 0.59 | 0.49 |
| MFA cash aid | | 9,525 | 0.23 | 0.42 | | 1,509 | 0.45 | 0.50 | | - | - | - |
| MFA subvention for SHI enrollment | |  |  |  | |  |  |  | |  |  |  |
| No subvention (reference) | | 8,527 | 0.50 | 0.50 | | - | - | - | | - | - | - |
| Partial subvention | | 8,527 | 0.25 | 0.43 | | - | - | - | | - | - | - |
| Full subvention | | 8,527 | 0.25 | 0.43 | | - | - | - | | - | - | - |
| Region | |  |  |  | |  |  |  | |  |  |  |
| Eastern region | | 9,537 | 0.48 | 0.50 | | 1,521 | 0.44 | 0.50 | | 11,570 | 0.47 | 0.50 |
| Central region | | 9,537 | 0.23 | 0.42 | | 1,521 | 0.26 | 0.44 | | 11,570 | 0.24 | 0.43 |
| Western region | | 9,537 | 0.18 | 0.39 | | 1,521 | 0.21 | 0.41 | | 11,570 | 0.19 | 0.39 |
| Northeastern region | | 9,537 | 0.11 | 0.31 | | 1,521 | 0.10 | 0.29 | | 11,570 | 0.10 | 0.30 |
| Log (total medical cost) | | 8,536 | 7.03 | 1.67 | | 1,521 | 8.19 | 1.08 | | 10,442 | 7.08 | 1.68 |
| Urban | | 9,537 | 0.61 | 0.49 | | 1,521 | 0.63 | 0.48 | | 11,570 | 0.63 | 0.48 |
| Number of males | | 9,537 | 1.47 | 0.91 | | 1,521 | 1.64 | 0.95 | | 11,570 | 1.52 | 0.92 |
| Number of older adults | | 9,537 | 0.56 | 0.75 | | 1,521 | 0.65 | 0.82 | | 11,570 | 0.56 | 0.75 |
| Number of children | | 9,537 | 0.08 | 0.31 | | 1,521 | 0.10 | 0.34 | | 11,570 | 0.09 | 0.33 |
| Adjusted household health score | | 9,520 | 3.78 | 2.06 | | 1,519 | 3.92 | 1.93 | | 11,551 | 3.59 | 1.96 |
| Number of people with chronic illnesses | | 9,525 | 0.76 | 0.82 | | 1,519 | 0.78 | 0.87 | | 11,557 | 0.78 | 0.83 |
| Number of people with serious illness | | 9,530 | 0.28 | 0.52 | | 1,521 | 1.14 | 0.38 | | 11,562 | 0.30 | 0.54 |
| Number of people needing long-term care | | 9,535 | 0.42 | 0.64 | | 1,521 | 0.70 | 0.71 | | 11,568 | 0.44 | 0.65 |
| Number of people with high school education and above | | 9,537 | 0.52 | 0.83 | | 1,521 | 0.64 | 0.89 | | 11,570 | 0.60 | 0.88 |
| Number of unemployed | | 9,537 | 0.78 | 0.82 | | 1,521 | 1.02 | 0.85 | | 11,570 | 0.80 | 0.82 |
| Log (per capita household income) | | 9,537 | 4.11 | 8.29 | | 1,521 | 4.20 | 8.18 | | 11,570 | 4.28 | 8.09 |

Table S7. Results of multilevel logistic models including the interaction between main independent variables and regions

|  | Regions | OR (95% CI) |
| --- | --- | --- |
| ***Model 1: Association between MFA subvention for SHI enrollment and SHI enrollment*** | | |
| Partial MFA subvention | Eastern region | 0.799 (0.508, 1.255) |
|  | Central region | 1.276 (0.792, 2.056) |
|  | Western region | 1.379 (0.908, 2.096) |
|  | Northeastern region | 1.116 (0.724, 1.721) |
| Full MFA subvention | Eastern region | 1.271 (0.971, 1.663) |
|  | Central region | 0.950 (0.803, 1.124) |
|  | Western region | 0.860 (0.488, 1.518) |
|  | Northeastern region | 1.456 (1.234, 1.718)*** |
| ***Model 2: Association between MFA cash aid and CHE*** | | |
| MFA cash aid | Eastern region | 1.060 (0.624, 1.801) |
|  | Central region | 1.027 (0.568, 1.858) |
|  | Western region | 0.868 (0.486, 1.547) |
|  | Northeastern region | 1.053 (0.750, 1.478) |
| ***Model 3: Association between SHI enrollment and CHE*** | | |
| Partial SHI enrollment | Eastern region | 0.892 (0.704, 1.129) |
|  | Central region | 0.873 (0.749, 1.018) |
|  | Western region | 0.991 (0.575, 1.709) |
|  | Northeastern region | 0.400 (0.155, 1.029) |
| Full SHI enrollment | Eastern region | 0.716 (0.583, 0.878)*** |
|  | Central region | 0.888 (0.817, 0.964)** |
|  | Western region | 0.733 (0.506, 1.063) |
|  | Northeastern region | 0.943 (0.302, 2.946) |

Notes: Odds ratio and 95% confidence interval of other covariates were not reported due to limited spaces. *=p<0.05, **=p<0.01, ***=p<0.001.

**References**

1. The Municipal Civil Affairs of Beijing. Opinions on adjusting the urban and rural Medical Financial Assistance scheme.2016. http://zhengwu.beijing.gov.cn/gzdt/gggs/t1358521.htm. Accessed 4 May 2016.
2. The Municipal Government of Tianjin. Opinions on improving the urban and rural Medical Financial Assistance scheme. 2009. http://www.tj.gov.cn/zwgk/wjgz/szfbgtwj/200908/t20090818_102024.htm. Accessed 4 May 2016.
3. The Municipal Government of Shijiazhuang, Hebei. Notification on improving the Medical Financial Assistance scheme of serious illness. 2015. http://info.hebei.gov.cn/hbszfxxgk/329975/329982/6500088/index.html. Accessed 4 May 2016.
4. The County Government of Yuanshi, Hebei. The Medical Financial Assistance scheme of Yuanshi. 2010. http://www.sjz.gov.cn/col/1275386943415/2010/12/02/1291251603166.html. Accessed 4 May 2016.
5. The Municipal Government of Hohhot, Inner Mongolia. The Medical Financial Assistance scheme of Inner Mongolia. 2005. http://www.nmgzfgb.gov.cn/information/nmgzb20/msg6770100963.html. Accessed 4 May 2016.
6. Inner Mongolia Daily (morning edition). The Medical Financial Assistance scheme of serious illness. 2013. http://www.nmgcb.com.cn/news/toutiao/2013/0717/40044.html. Accessed 4 May 2016.
7. The District Government of Hohhot, Inner Mongolia. Notification on improving the Medical Financial Assistance scheme of Xincheng district. 2013. http://www.hhxc.gov.cn/xxgk/zfxxgkml/41a2e03e-7a57-4340-b679-a2a1009c2653.html. Accessed 4 May 2016.
8. Li X, Munila, Qin J, Tang J, Mao L. Xinjiang Medical Financial Assistance scheme status study. Soft Science Health. 2010; 24(06):557-59.
9. The Administration Bureau Office of Kashi, Xinjiang. Notification on improving the Medical Financial Assistance scheme of Kashi. 2009. http://www.lawyee.net/Act/Act_Display.asp?RID=710378. Accessed 4 May 2016.
10. The Municipal Government of Xinin, Qinghai. The Medical Financial Assistance scheme of Qinghai. 2010. http://www.qhcl.org/html/17/2158.html. Accessed 4 May 2016.
11. The Municipal Government of Lanzhou, Gansu. The Medical Financial Assistance scheme of Gansu. 2010. http://baike.baidu.com/link?url=1cc0OgQXLtWiQdlHC_R0Q4qIg8UX-0Iab8VQ4zPV9u-Ofi1anZIHJwINwRrgjOH6jlfAT1qdknSffsqxlcj4Nq. Accessed 4 May 2016.
12. The Municipal Government of Lanzhou, Gansu. The Medical Financial Assistance scheme of Lanzhou. 2014. http://mzj.lanzhou.gov.cn/zcfg/201501/t20150123_375423.html. Accessed 4 May 2016.
13. The Municipal Government of Yinchuan, Ninxia. The Medical Financial Assistance scheme of Ninxia. 2015. http://www.pkulaw.cn/fulltext_form.aspx?Gid=17916330. Accessed 4 May 2016.
14. The Municipal Government of Chengdu, Sichuan. Notification on improving urban Medical Financial Assistance of Chengdu. 2015. http://www.qionglai.gov.cn/index.php?cid=245&tid=44779. Accessed 4 May 2016.
15. The Municipal Government of Chongqing. Opinions on improving urban and rural Medical Financial Assistance scheme of Chongqing. 2012. Accessedhttp://www.wzmzw.gov.cn/sub/201303/4631.shtml. Accessed 4 May 2016.
16. The Municipal Government of Xian, Shaanxi. The urban and rural Medical Financial Assistance scheme of Shaanxi. 2014. http://shaanxi.mca.gov.cn/article/mzyw/jzgl/201404/20140400617614.shtml. Accessed 4 May 2016.
17. The Civil Affairs Bureau of Yulin, Shaanxi. Notification on raising Medical Financial Assistance standard of Yulin. 2014. http://www.hszf.gov.cn/jiuzhuzhengcewenjian/2155.jhtml. Accessed 4 May 2016.
18. The Municipal Government of Taiyuan, Shanxi. The Medical Financial Assistance scheme of Shanxi. 2013. http://www.shanxigov.cn/n16/n8319541/n8319687/n8327404/17446859.html. Accessed 4 May 2016.
19. The Municipal Government of Zhengzhou, Henan. Draft decree on Social Assistance of Henan. 2014. http://luoyang.mca.gov.cn/article/zcwj/201501/20150100765118.shtml Accessed 4 May 2016.
20. The Municipal Government of Luoyang, Henan. Notification on improving urban and rural Medical Financial Assistance scheme of Luoyang. 2014. http://luoyang.mca.gov.cn/article/mzyw/yljz/zcfg/201407/20140700671047.shtml. Accessed 4 May 2016.
21. The Municipal Government of Hefei, Anhui. The Medical Financial Assistance scheme of Anhui. 2015. http://www.mengcheng.gov.cn/openness/detail/content/54c6ec9a7f8b9a5a6fe04bab.html. Accessed 4 May 2016.
22. The Municipal Government of Hefei, Anhui. The Medical Financial Assistance scheme of Hefei. 2014. http://wcwy.ahxf.gov.cn/village/s4newcontent.asp?WebID=5433&Class_ID=186826&id=1547196. Accessed 4 May 2016.
23. The Municipal Government of Nanchang, Jiangxi. Notification on improving Medical Financial Assistance scheme of Jiangxi. 2015. http://www.jiangxi.gov.cn/zzc/ajg/sbgt/201601/t20160114_1242622.htm. Accessed 4 May 2016.
24. The Department of Civil Affairs of Shandong Province. The urban and rural Medical Financial Assistance scheme of Shandong. 2012. http://www.sdmz.gov.cn/articles/ch00188/201209/d96d5012-56b7-402d-b9f4-98aae1412ca8.htm. Accessed 4 May 2016.
25. The Municipal Government of Jinan, Shandong. The urban and rural Medical Financial Assistance scheme of Jinan. 2011. http://www.jinan.gov.cn/art/2011/12/14/art_1773_53619.html. Accessed 4 May 2016.
26. The Municipal Government of Guangzhou, Guangdong. The urban and rural Medical Financial Assistance of Guangdong. 2012. Accessedhttp://www.gdqy.gov.cn/gdqy/shbz/201211/e031f587e4be4e32a4189415bbd15fb7.shtml. Accessed 4 May 2016.
27. The Municipal Government of Guangzhou, Guangdong. The Medical Financial Assistance scheme of Guangzhou. 2016. http://sfzb.gzlo.gov.cn/sfzb/file.do?fileId=2C9089255285EFDB01528B1A06DB000C. Accessed 4 May 2016.
28. The Municipal Government of Fuzhou, Fujian. Opinions on improving urban and rural Medical Financial Assistance scheme. 2016. http://www.fujian.gov.cn/zc/zwgk/zxwj/szfwj/201602/t20160202_1136563.htm. Accessed 4 May 2016.
29. The Municipal Government of Fuzhou, Fujian. The Medical Financial Assistance scheme of Fuzhou. 2009. http://www.fjsn.gov.cn/shouning/html/info/article/1164/3345.shtml. Accessed 4 May 2016.
30. The Municipal Government of Nanning, Guangxi. The urban and rural Medical Financial Assistance scheme of Guangxi. 2012. http://longlin.mca.gov.cn/article/zcwj/201208/20120800344034.shtml. Accessed 4 May 2016.
31. The Municipal Government of Baise, Guangxi. Notification on improving urban Medical Financial Assistance scheme of Baise. 2012. http://baise.mca.gov.cn/article/zcwj/201301/20130100414696.shtml. Accessed 4 May 2016.
32. The Municipal Government of Longling, Yuannan. The urban and rural Medical Financial Assistance scheme of Longling. 2015. http://www.longling.gov.cn/zwgw/7622934289258426318. Accessed 4 May 2016.
33. The Municipal Government of Haikou, Hainan. Draft decree on Medical Financial Assistance of Hainan. 2009. http://baike.baidu.com/link?url=MVK29EvqZSElsrauX4lIS5brA_qU-FcB97uA5C4C9amMmagBL0q3Y3Xiqa9W8Pm0cRL6nT1NUcbdCbs2maYgrU3W7p30DVE_YSXBWt0HKHy3g2AYkfAB0pTyL9XokCO7xHdnpAWYyHLQd1XNv4kCnTLGlkcxdpwGkkcHAhoiBIyh1cD9AdXQUXnaikLcT_21mSOrIZTlOPQDjpUF1e2fvkV9Sc1T1fL3Hpu7Arrejo-HANGEVpmbB_XOm4wpXVt7. Accessed 4 May 2016.
34. The Municipal Government of Haikou, Hainan. Application instructions of Medical Financial Assistance in Haikou. 2010. Accessedhttp://www.hainan.gov.cn/hn/wsbs/ggfw/yl/fwlb/ylbz/yljz/201211/t20121127_804281.html. Accessed 4 May 2016.
35. The Municipal Government of Shenyang, Liaoning. Opinions on improving Medical Financial Assistance scheme of Liaoning. 2014. http://www.pkulaw.cn/fulltext_form.aspx?Gid=17658953. Accessed 4 May 2016.
36. The Municipal Government of Changchun, Jilin. Opinions on improving Medical Financial Assistance scheme of Jilin. 2015. http://www.mca.gov.cn/article/zwgk/dfxx/201509/20150900873662.shtml. Accessed 4 May 2016.
37. Heilongjiang Daily. Heilongjiang introduced urban and rural Medical Financial Assistance regulations to improve the Medical Financial Assistance. 2016. http://heilongjiang.dbw.cn/scheme/2016/01/10/057031273.shtml. Accessed 4 May 2016.
38. The Municipal Government of Harbin, Heilongjiang. The urban and rural Medical Financial Assistance scheme of Harbin. 2009. http://zwgk.harbin.gov.cn/auto336/auto344/200911/t20091105_36430.html. Accessed 4 May 2016.
39. The Municipal Government of Hangzhou, Zhejiang. Notification on improving Medical Financial Assistance scheme of Zhejiang. 2014. http://www.zh.gov.cn/zwgk/fggw/szcfg/201501/t20150112_350436.shtml. Accessed 4 May 2016.
40. The Municipal Government of Nanjing, Jiangsu. Notification on improving Medical Financial Assistance scheme of Nanjing. 2016. http://www.nanjing.gov.cn/njszfnew/szf/201602/t20160222_3793177.html. Accessed 4 May 2016.
41. The Municipal Government of Shanghai. Notification on adjusting and improving Medical Financial Assistance scheme of Shanghai.2015. http://www.shmzj.gov.cn/gb/shmzj/node687/u1ai41198.html. Accessed 4 May 2016.
42. The Municipal Government of Changsha, Hunan. The urban Medical Financial Assistance scheme of Changsha. 2015. http://www.changsha.gov.cn/xxgk/szfxxgkml/zfgb/2015n/201506_28194/201507/t20150721_789449.html. Accessed 4 May 2016.
43. The Municipal Government of Wuhan, Hubei. The urban Medical Financial Assistance scheme of Wuhan. 2013. http://www.whmzj.gov.cn/News_View.aspx?id=1629. Accessed 4 May 2016.
44. The Municipal Government of Guiyang Guizhou. Handbook of urban and rural Medical Financial Assistance policy of Guizhou. 2013. http://www.gzgov.gov.cn/zxfw/ylfw/yljz/201506/t20150630_259544.html. Accessed 4 May 2016.
